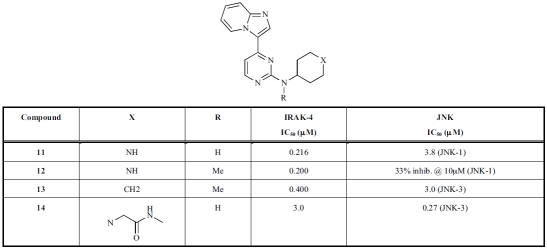# IRAK-4 Inhibitors for Inflammation

**DOI:** 10.2174/156802609789044407

**Published:** 2009-05

**Authors:** Zhulun Wang, Holger Wesche, Tracey Stevens, Nigel Walker, Wen-Chen Yeh

**Affiliations:** 1 Amgen Inc., 1120 Veterans Boulevard, South San Francisco, CA 94080; 2 Amgen Inc., 1201 Amgen Court West, Seattle, WA 98119

**Keywords:** Interleukin-1 receptor-associated kinase (IRAK), IRAK-4 inhibitors, kinase inhibitors, binding mode, X-ray crystal structure.

## Abstract

Interleukin-1 receptor-associated kinases (IRAKs) are key components in the signal transduction pathways utilized by interleukin-1 receptor (IL-1R), interleukin-18 receptor (IL-18R), and Toll-like receptors (TLRs). Out of four members in the mammalian IRAK family, IRAK-4 is considered to be the “master IRAK”, the only family member indispensable for IL-1R/TLR signaling. In humans, mutations resulting in IRAK-4 deficiency have been linked to susceptibility to bacterial infections, especially recurrent pyogenic bacterial infections. Furthermore, knock-in experiments by several groups have clearly demonstrated that IRAK-4 requires its kinase activity for its function. Given the critical role of IRAK-4 in inflammatory processes, modulation of IRAK-4 kinase activity presents an attractive therapeutic approach for the treatment of immune and inflammatory diseases. The recent success in the determination of the 3-dimensional structure of the IRAK-4 kinase domain in complex with inhibitors has facilitated the understanding of the mechanistic role of IRAK-4 in immunity and inflammation as well as the development of specific IRAK-4 kinase inhibitors. In this article, we review the biological function of IRAK-4, the structural characteristics of the kinase domain, and the development of small molecule inhibitors targeting the kinase activity. We also review the key pharmacophores required for several classes of inhibitors as well as important features for optimal protein/inhibitor interactions. Lastly, we summarize how these insights can be translated into strategies to develop potent IRAK-4 inhibitors with desired properties as new anti-inflammatory therapeutic agents.

## INTRODUCTION AND BIOLOGY

Chronic inflammatory disorders and autoimmune diseases are critical health problems that require therapeutic interventions and affect the lives of millions of people worldwide. In addition, at least some components of other common diseases, for example, type 2 diabetes and cardiovascular diseases, are associated with deregulation of inflammatory signals. Understanding the intricately regulated nature of inflammatory modulation and the complex signaling systems involved is the key to devising potential therapeutic strategies. As multiple cytokines, mediators, and signaling pathways are involved in various inflammatory disorders, one of the main challenges is to identify key target(s) that impact the most relevant pathways.

Interleukin-1 Receptor-Associated Kinase 4 (IRAK-4) [1] is an essential signal transducer downstream of interleukin-1 receptor (IL-1R), interleukin-18 receptor (IL-18R), and Toll-like receptors (TLRs). All these receptors contain a conserved intracellular Toll and interleukin-1 receptor (TIR) domain [2]. TLRs receive signals from ligands that are mainly derived from or in reaction to different microorganisms or endogenous stimuli and initiate the first-wave of inflammatory signals and innate immune responses [3, 4]. Therefore, TLRs play a key role in many disease processes, including response to infections and various auto-inflammatory disorders, and are implicated in many other human diseases [5]. IL-1, like tumor necrosis factor-α (TNF-α) and other major cytokines, is a key contributor to the inflammation network that propagates and amplifies signals. Furthermore, signaling pathways mediated by TLRs, IL-1R, and other cytokine receptors may communicate in various cross-talk mechanisms. Therefore, a key signaling molecule downstream of IL-1R and TLRs would have profound effects on overall inflammatory responses and may be an effective therapeutic target for various diseases associated with deregulated inflammation.

Three lines of evidence suggest that the presence of IRAK-4 is absolutely required for TIR signaling. Firstly, in MyD88-dependent TIR signaling, IRAK-4 is the initial receptor-proximal protein kinase that is recruited and activated upon ligation of receptors, and it interacts with multiple, key downstream signaling molecules [1]. Secondly, targeted deletion of IRAK-4 in mice revealed severe defects in cytokine responses and downstream signaling pathways induced by IL-1R and TLRs [6]. Thirdly, human patients who are deficient in IRAK-4 due to gene deletion/mutations have been described [7]. Cells derived from these patients clearly exhibit impaired responses to IL-1, IL-18, or exogenous MyD88-dependent TLR ligands [7]. Together with its broad impact on TLR and IL-1R signaling, the evidence collectively suggests that IRAK-4 may be a key target for therapeutic intervention in various inflammatory disorders.

One obvious question that follows is whether inhibition of IRAK-4 may cause too broad an impact, rendering the side effects intolerable, given the molecule’s central position in multiple signaling pathways. Children with IRAK-4 deficiency are indeed susceptible to certain pyogenic infections, particularly *Streptococcus*, but no severe viral or parasitic infections have been reported [7, 8]. Despite the critical role that IRAK-4 plays in TIR signaling, adult patients with the deficiency are not prone to chronic infections. In fact, as these patients approach adolescence, susceptibility to infections becomes increasingly rare [9, 10]. It is possible that modulation of IRAK-4 function through kinase inhibition, for example, may provide therapeutic benefits by toning down inflammatory responses, while protective immunity remains sufficiently preserved to protect against microbial infections.

**Fig. (1):**
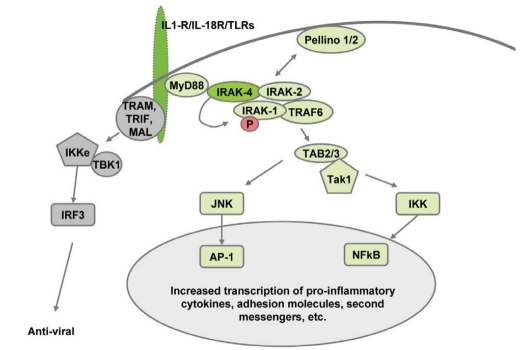
TIR signaling pathways. This figure illustrates that inhibition of IRAK-4 kinase activity should primarily block MyD88-dependent TLR signaling, resulting in induced AP-1 and NF-κB activation, while anti-viral responses should remain mainly intact.

IRAK-4 belongs to a family of mammalian IRAKs that include IRAK-1 [11], IRAK-2 [12], and IRAK-M, also known as IRAK-3 [13]. All four IRAK family members appear to play a role in Toll and IL-1R signaling [14-16]. They share a similar domain structure containing a conserved N-terminal death domain (DD) and a central kinase domain (KD). Upon stimulation of IL-1R, IL-18R, and most TLRs, MyD88 is the first adaptor protein which binds to the receptors and is followed by recruitment of IRAK-4 and activation of its kinase activity (Fig. **1**). IRAK-4 transduces inflammatory signals by rapidly activating IRAK-1. This is followed by the activation of IRAK-2, which has recently been shown to play a key role in sustaining pro-inflammatory cytokine production [17]. Signals are then relayed to the TRAF6 protein complex, facilitating downstream signaling events. IRAK-M appears to play a negative regulatory role in this process [18]. Mouse knock-out studies have demonstrated an essential role for IRAK-4 in IL-1R, IL-18R, and most TLR signaling [6], while IRAK-1-deficient mice and cells show partial defects or defects in more specific pathways [19-21]. Further supporting its essential function, IRAK-4 shares the highest homology with the *Drosophila* Pelle protein, an ortholog of mammalian IRAKs. Pelle plays a critical role in the *Drosophila* Toll signaling pathway and requires its kinase activity for signal transduction [22].

IRAK-4 knock-out mice are severely impaired in signaling and cellular responses to IL-1, IL-18, and most TLR ligands. IRAK-4-mediated signals are essential for downstream activation of JNK, NF-κB, and p38 MAPK [6, 23], all of which play a role in cytokine and inflammatory responses. However, it is worth noting that certain TLRs also mediate signals to activate the IRF family of transcription factors that lead to induction of additional genes, including type I interferons [4, 24]. Different TLRs may recruit distinct MyD88 family members of adaptors and activate different IRFs [4]. Among these, IRAK-4 appears to only play a role in the activation of IRF5 and IRF7 mediated through TLR7 and TLR9 [25-27] but not in other pathways leading to IRF and type I interferon responses. Studies with IRAK-4-deficient patients have demonstrated reduced interferon-α (IFN-α) and IFN-β production in response to TLR ligands while responses to herpes simplex virus (HSV) and vesicular stomatitis virus (VSV) remained intact [28]. The involvement of IRAK-4 in TLR7 and TLR9 signaling, coupled with the observation that dual inhibition of TLR7 and TLR9 in lupus-prone mice results in amelioration of disease symptoms, indicates that IRAK-4 may be a suitable therapeutic target for systemic lupus erythematosus (SLE) [26, 29].

IRAK-4 may transduce signals through physical protein-protein interaction and through its kinase activity, which activates downstream molecules such as IRAK-1 [1]. It is therefore critical to examine if IRAK-4 kinase activity is essential for its signaling functions. Initial studies using biochemical approaches, over-expression experiments, and reconstitution of IRAK-4 knock-out cells with kinase inactive mutants all point to the requirement of IRAK-4 kinase activity for its signal transduction [1, 30]. At a minimum, specific pathways such as IL-1-induced NF-κB and JNK that were examined in these systems required IRAK-4 kinase functions. However, cells expressing only an IRAK-4 kinase inactive mutant were still able to respond to IL-1 in NF-κB activation and cytokine production, although the response was greatly reduced compared to wild type [30]. Another study utilizing IRAK-4 mutant variants identified from human patients demonstrated that IRAK-4 with a truncated kinase domain inhibited IL-1 signaling by disrupting formation of the receptor complex [8]. 

Several recent publications using different strains of IRAK-4 kinase-dead mutant knock-in mice further confirm the importance of IRAK-4 kinase activity [23, 31-34]. In essence these knock-in mice and cells derived from these mice express only IRAK-4 kinase inactive mutant, a mutation of the conserved residues in the ATP binding pocket, and no wild type IRAK-4. While there are some variations of the experiments and findings among different knock-in strains, these mutants collectively demonstrate substantial defects in signaling pathways and cytokine induction in response to IL-1 and various TLR ligands. These signaling and cytokine defects observed in knock-in mutants appear similar to those observed in IRAK-4 knock-out mice [23, 33, 34]. 

All of these data suggest that IRAK-4 may be a suitable target for inflammatory diseases involving multiple receptors including IL-1R (rheumatoid arthritis (RA) and osteoarthritis (OA) [35]), IL-18R (inflammatory bowel disease (IBD) [36, 37]), TLR2, 4 (RA [38]), and TLR7, 9 (SLE [26, 29]). 

## STRUCTURAL FEATURES OF THE IRAK-4 KINASE DOMAIN 

The recent success in the determination of the three dimensional structures of the IRAK-4 kinase domain by X-ray crystallography has provided great insights into IRAK-4’s mechanistic role in immunity and inflammation, its inhibitor binding, and the design of selective inhibitors as potential therapeutics. To date, six crystal structures of the IRAK-4 kinase domain have been deposited with the RCSB Protein Data Bank (PDB) (Table **1**); two of them are solved in the apo form, one in complex with a non-hydrolysable ATP analogue AMP-PNP, two in complex with the generic kinase inhibitor staurosporine, and one in complex with a synthetic inhibitor derived from a medicinal chemistry effort [39, 40].

**Fig. (2):**
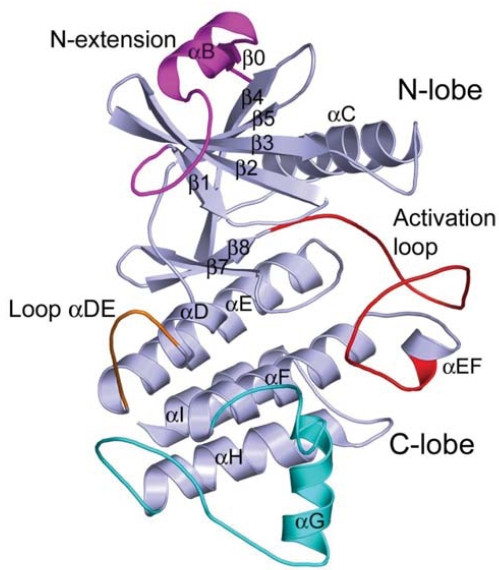
Overall structure of the IRAK-4 kinase domain (PDB code: 2NRU).

The overall structure of the IRAK-4 kinase domain adopts a canonical fold of classical protein kinases, with the ATP-binding cleft sandwiched between a bilobal arrangement (Fig. **2**). The N-terminal lobe (N-lobe) consists mainly of a twisted five-stranded antiparallel β-sheet and one α helix (αC), and the larger C-terminal lobe (C-lobe) is predominantly α-helical. Structural comparison of IRAK-4 kinase with other serine/threonine or tyrosine kinases shows that most of the conserved core structural elements are in good alignment. Yet, the structure reveals a few unique features for IRAK-4 kinase, including an additional α helix from the N-terminal extension in the N-lobe, a longer loop between helices αD and αE (residues 276-282, loop αDE), and a significantly moved helix αG (residues 395-406) as well as its adjoining loops (residues 383-394 and 407-423).

### N-Terminal Extension 

The IRAK-4 protein kinase constructs that yielded crystal structures have extra ordered residues extending from the normal N-terminus of the kinase domain. This N-terminal extension was found to be important for protein expression as well as kinase activity when compared with the full length protein [39]. Many other protein kinase structures also reveal such N-terminal extensions. While these extensions share no sequence homology or structural similarity, they have the common characteristic of packing tightly against the β-sheet and becoming an integral part of the overall fold [41]. In the case of IRAK-4, the N-terminal extension forms a short extra β-strand packed in parallel with the β4 strand, an extra α-helix (αB), and then a loop. The helix (αB) crosses over the five-stranded β-sheet of the N-lobe and makes hydrophobic interactions with the concave hydrophobic surface of the β-sheet. The following loop region is described as having three helix-capping motifs [40], including an ST motif (residues 177-181) [42], an ASX motif (residues 181-183) [43], and a solvent-exposed Schellman motif (residues 184-189) [44] characterized by hydrogen bonding interactions between the backbone amines of Gly188/Gly189 and the backbone carbonyls of Pro184/Ile185.

**Fig. (3):**
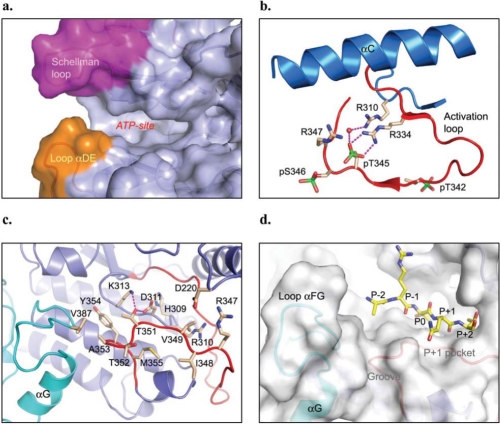
Unique features of the IRAK-4 kinase domain. **a)** The Schellman loop in the N-terminal extension (shown in magenta) and the extra long loop αDE (shown in orange) extends the ATP-binding pocket (PDB code: 2NRU). **b)** Activation loop of IRAK-4 with its phosphate ligand binding interactions resembling that of protein tyrosine kinases (PDB code: 2OIB). **c)** The P+1 site of IRAK-4 with a conserved threonine residue in serine/threonine kinases, Thr315, interacting with the catalytic base Asp311 and Lys313 (PDB code: 2NRU). **d)** The substrate-binding area of IRAK-4, docked with a putative substrate peptide from the activation loop of IRAK-1 (shown in yellow sticks).

Interestingly, while the Schellman loop points down to the C-lobe into the cleft region between the two lobes, the loop αDE sticks out of the C-lobe and points up to the N-lobe in the cleft region as well. Together, the Schellman loop and the extra long loop αDE provide an extended protein surface area for the ATP binding site in IRAK-4 (Fig. **3a**).

### Activation Loop

Regulation of IRAK-4 kinase activity has been reported by the autophosphorylation of three sites in the activation loop, i.e., Thr342, Thr345, and Ser346 [45]. All the reported IRAK-4 kinase domain structures are solved in the phosphorylated forms, although the phosphorylation states vary among them (Table **1**). The conformation of the phosphorylated activation loop of IRAK-4 is, in general, similar to that of other phosphorylated serine/threonine kinases such as cAPK [46], cyclin-dependent kinase CDK2 [47], and MAP kinase ERK2 [48] as well as tyrosine kinases such as LCK [49] and insulin receptor kinase (IRK) [50]. pSer346 points outward to the solvent and its phosphate group makes no contacts with protein. pThr342 is positioned away from the catalytic site with its phosphate moiety coordinated by the side chains of Lys367 of helix αF and Lys441 of helix αEF. pThr345 is folded back towards the catalytic center in the protein core with its phosphate group positioned to compensate for a positively charged pocket formed by three arginine residues, i.e., Arg310 that precedes the catalytic base Asp311 in the catalytic loop, Arg334 that is the third residue following the signature DFG motif upstream of the activation loop, and Arg347, which is also in the activation loop but is downstream of the two phosphorylated residues. The phosphate group of pThr345 is coordinated directly to Arg334 (Fig. **3b**). Additional interactions with the phosphate group of pThr345 are a water-mediated hydrogen bond with Arg310 of the catalytic loop and a direct hydrogen bond to the backbone amide nitrogen of Ser346. Here, the structures suggest that pThr345 is the prototypical phosphoresidue responsible for the activation of IRAK-4 kinase. The phosphorylations of Thr342 and Ser346 might play roles in stabilization of the activation loop in the active state.

Interestingly, the phosphate interaction pattern of IRAK-4 kinase differs from that of other phosphorylated serine/ threonine protein kinases, where two or three phosphate-binding residues are normally observed [46, 48, 51]. Among these positively charged residues, the ones from the catalytic loop and helix αC are believed to be important for promoting the correct orientation and electrostatic environment for the catalytic base Asp311 as well as the proper active orientation for helix αC [52]. Instead, the pattern of the phosphate ligand binding interactions of IRAK-4 resembles closely those of tyrosine protein kinases, such as LCK [49], IRK [50], insulin-like growth factor 1 receptor kinase (IGF1RK) [53], and more recently Janus Kinase 3 (JAK3) [54]. All of these kinases share a common motif for the phosphate ligand binding interaction, i.e. with a single primary phosphate ligand arginine or lysine in the activation loop, in an equivalent position to Arg334 of IRAK-4. In fact, the phosphate interaction pattern of IRAK-4 is almost identical to that of protein tyrosine kinase IRK, although IRAK-4 is a serine/threonine protein kinase. 

### Substrate-Binding Site

In comparison with other protein kinases, IRAK-4 kinase also presents a unique substrate-binding site. The fold of the P+1 pocket of IRAK-4, defined by the downstream surface loop of the activation loop to helix αEF (residues I^348^VGTTAYM^355^), closely resembles those in cAPK and PhK, with a serine/threonine kinase conserved threonine residue Thr351 (corresponding to Thr186 in PhK) interacting with catalytic base Asp311 and Lys313 from the catalytic loop (Fig. **3c**). Yet, there is a substantial difference in the nature and size of the binding site in IRAK-4. Instead of being lined with all hydrophobic residues in the P+1 pocket, such as Val183, Pro187, and Leu190 in PhK, and Leu198, Pro202, and Leu205 in cAPK, IRAK-4 has Ile348, Thr352, and Met355. As a result, IRAK-4 substrates are likely to adopt a similar conformation around the P+1 site, but with different specificity. A large and more polar P+1 residue is preferred to interact with Thr352 in the P+1 loop. Moreover, two charged residues, Arg347 from the activation loop and Asp220 from the loop following helix αC, reach the P+2 site. Thus, a polar or charged P+2 residue is preferred for the IRAK-4 substrate. 

The IRAK-4 structure presents a distinctive change in the region of helix αG which moves significantly away from the active site and helix αEF, with about a 10 Å shift as compared with other protein kinases such as tyrosine kinase LCK[49, 55] and serine/threonine kinase cAPK [46]. Such a dramatic movement of the helix αG and its flanking loops has a direct impact on the substrate-binding site of IRAK-4. The shift made by helix αG in IRAK-4 offers an open space at the side of the P+1 pocket and creates a large hydrophobic groove bound by the region of the C-terminal activation loop, the P+1 loop, and helix αG (Fig. **3d**). Interestingly, this hydrophobic groove is typically the location for helix αG of other protein kinases. While helix αG shifts away from the P+1 site, the loop preceding helix αG actually shifts closer to the P+1 loop with Val387 making van der Waals contact with Ala353 in the P+1 loop (Fig. **3c**). In comparison with PhK, where the P-2 residue from substrate peptide approached this region, a large hydrophobic residue is likely preferred at P-2 for the IRAK-4 substrate.

### ATP-Binding Site and Tyrosine Gatekeeper

The ATP-binding site between the two lobes of protein kinases has been the target for small molecule inhibitors to modulate kinase activities. Elucidation of unique characteristics in the ATP-pocket of each individual kinase becomes a must for successfully designing potent and selective inhibitors. While IRAK-4 shows a similar overall core structure for the ATP binding site compared with other protein kinases, a pivotal residue at the center of the ATP-binding site, commonly known as a “gatekeeper”, is unique for IRAK-4. The term “gatekeeper” refers to a residue upstream of the hinge loop that connects the two lobes. The gatekeeper residue controls access to a pre-existing internal hydrophobic pocket at the back of the ATP-binding site that nucleotides do not exploit [56]. While nearly 20% of kinases in the human kinome possess a smaller threonine residue as a gatekeeper, 40% of them have a large methionine residue, and almost 15% of them have a large phenylalanine residue. IRAK-4 has a tyrosine residue, Tyr262, at this position. A tyrosine gatekeeper seems to be exclusive to the IRAK family of kinases when compared with a limited set of more than 400 kinases in the human kinome database. IRAK-1, IRAK-2, and IRAK-M also have a tyrosine as the gatekeeper residue.

**Fig. (4):**
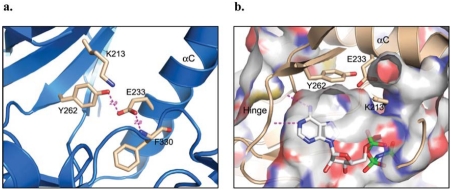
The gatekeeper region and the ATP-binding pocket of IRAK-4. **a)** The unique Tyr262 gatekeeper interacts with an absolutely conserved glutamate residue Glu233, disrupting the usual Glu-Lys salt bridge (PDB code: 2OIB). **b)** The ATP-binding site has no back pocket due to the tyrosine gatekeeper (PDB: 2OID).

The gatekeeper residue has been shown to play a critical role in controlling kinase selectivity for various small molecule ATP-competitive inhibitors. The back hydrophobic pocket is more readily probed by a diverse class of inhibitors for protein kinases with a smaller gatekeeper residue like threonine. A bulkier gatekeeper, such as methionine or phenylalanine, seen for the majority of protein kinases, makes the entrance to this pocket relatively narrow. Here, with tyrosine at the gatekeeper position and its hydroxyl group forming a direct hydrogen bond interaction with one of the carboxylate oxygen atoms of the conserved glutamate Glu233 from helix αC (Fig. **4a**), the back hydrophobic pocket of the ATP binding site is completely blocked for access in IRAK-4 (Fig. **4b**).

**Fig. (5):**
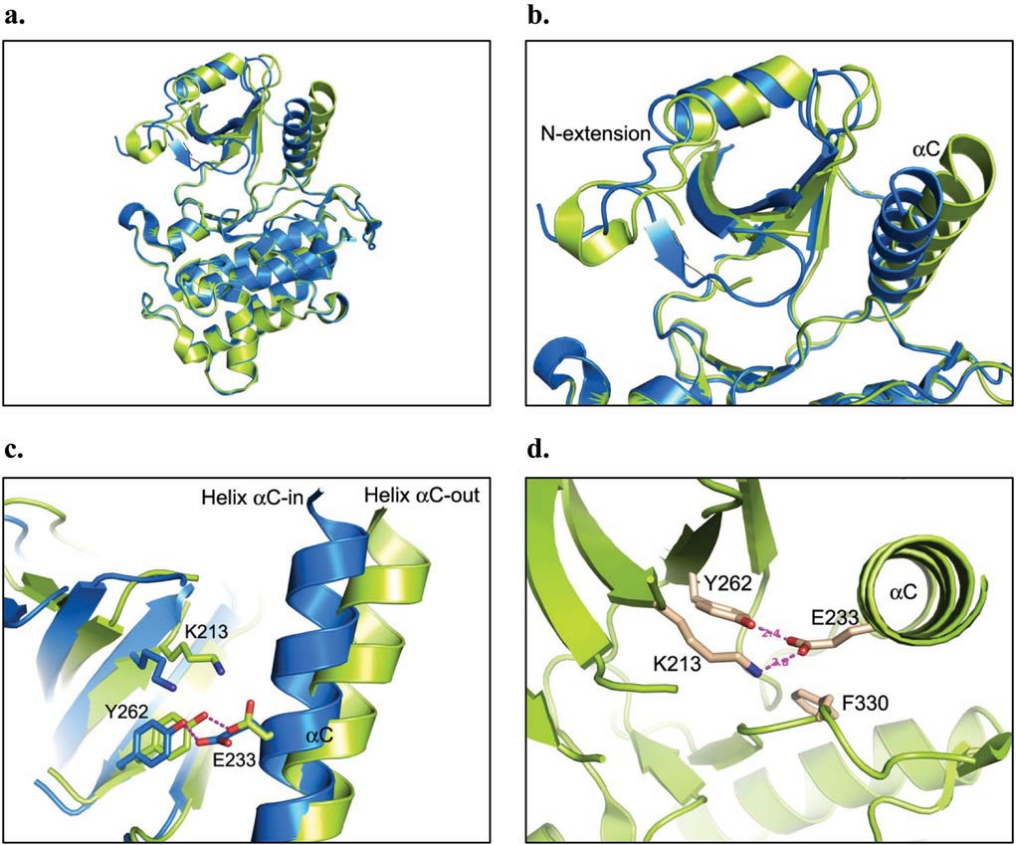
The dual conformations of apo IRAK-4 (PDB code: 2OIB). **a)** Overall two conformations of IRAK-4. **b)** Two conformations differ mainly in the N-lobe where helix αC, the Gly-rich loop, and the N-terminal extension show evident movements. **c)** Two conformations are symbolized by “helix αC-in” and “helix αC-out” positions, where the invariant Glu233 shows different side chain conformations. **d)** The usual Glu-Lys salt bridge is observed only in the “helix αC-out” conformation.

In the apo crystal structure of IRAK-4 reported by Kuglstatter *et al*. (PDB code: 2OIB) [40], two distinct protein conformations are observed in the four independent protein molecules in the crystallographic asymmetric unit. The two conformations vary mainly in their relative orientation between the N- and C-lobes, with helix αC, the Gly-rich loop, and the N-terminal extension experiencing the most evident movements (Figs. **5a**, **5b**). These two conformations are referred to as “helix αC-in” and “helix αC-out” [40].

Conformational changes in helix αC have been described as one of the signature features of the kinase activation mechanism for both serine/threonine and tyrosine kinases [57]. Upon kinase activation, as a coupled action of the activation loop phosphorylation, helix αC undergoes a conformational change by moving into the catalytic center and facilitating the salt bridge between the invariant catalytic lysine residue and the conserved glutamate residue on helix αC. Hence, “helix αC-in and αC-out” conformations are normally associated with kinase activity states, with “helix αC-in” and the lysine-glutamate salt-bridge representing the active form of kinases. In IRAK-4, tyrosine gatekeeper Tyr262, which acts as a principal partner for the invariant glutamate Glu233 of helix αC, interferes with this classical ion-pair interaction. In both observed conformations of IRAK-4, the gatekeeper Tyr262 forms direct hydrogen bond interactions with Glu233 (Fig. **5c**). Interestingly, the lysine glutamate salt bridge is observed only in the “helix αC-out” conformation for IRAK-4 (Fig. **5d**). Here, the glutamate Glu233 forms hydrogen bond interactions with both the catalytic Lys213 and the gatekeeper Tyr262.

The different protein conformations are also observed in other solved IRAK-4 structures. However, only one conformations was usually observed for each of the structures. In the other deposited apo structure (PDB code: 2O8Y), both independent protein molecules were crystallized in the “helix αC-in” conformation. In the cocrystal structure with AMP-PNP (PDB code: 2OID), the protein molecules mainly assume the “helix αC-in” conformation. While the protein molecules adopt primarily the “helix αC-out” conformation in the staurosporine cocrystal structures (PDB code: 2NRY and 2OIC), all four independent protein molecules take up the “helix αC-in” conformation in the IRAK-4 cocrystal structure with a benzimidazole synthetic inhibitor (PDB code: 2NRU). Apparently, both protein conformations coexist in solution [40]. Since the “helix αC-in” conformationsis associated with the AMP-PNP bound form, this form was proposed to represent the active form of the kinase [40].

In summary, the structural data revealed that IRAK-4, although characterized as a serine/threonine kinase, contains characteristic structural features of both serine/threonine and tyrosine kinases, suggesting the possibility that IRAK-4 is a dual specificity kinase. This finding provides valuable insight into a better understanding of the role of IRAK-4 in both innate and acquired immunity. In addition, IRAK-4 offers a few unique features when compared with other protein kinases, especially the tyrosine gatekeeper and the extended ATP binding area, which have a direct impact on inhibitor design.

## IRAK-4 INHIBITOR DEVELOPMENT

**Fig. (6):**
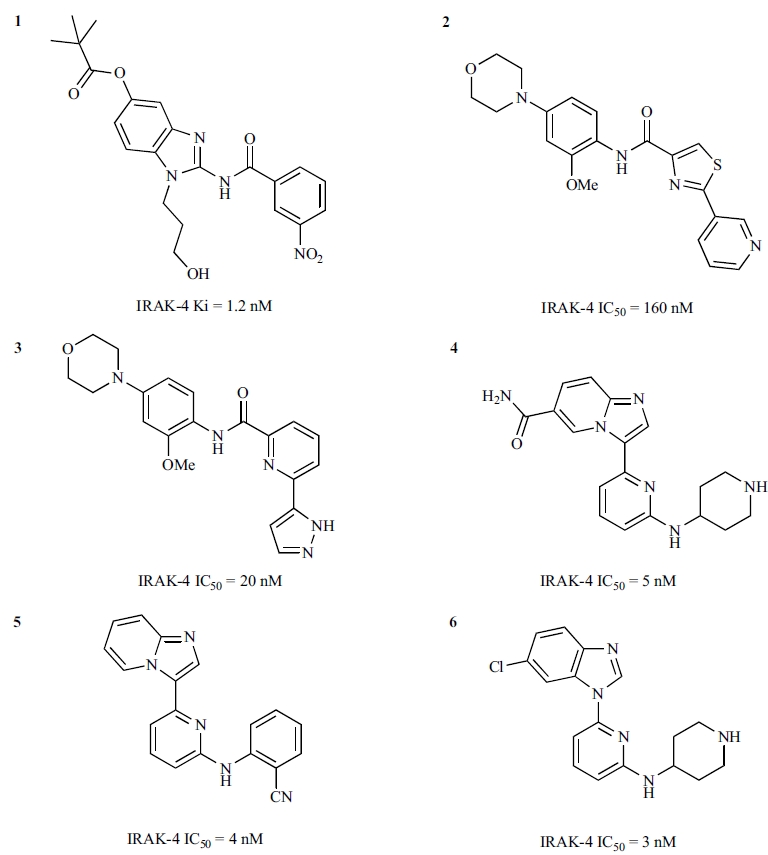
Chemical structures of representative IRAK-4 inhibitors discovered through medicinal chemistry efforts.

Potent and selective small molecule inhibitors targeting IRAK-4 kinase activity are currently being pursued as potential therapeutics for autoimmune and inflammatory diseases [39, 58-64]. A few novel series of potent IRAK-4 kinase inhibitors have been described, including amino-benzimidazoles [59], thiazole or pyridine amides [60], imidazo[1,2-*a*]pyridines [61, 62], imidazo[1,2-*b*]pyridazines, and benzimidazole-indazoles [58, 64]. The chemical structures of some representative inhibitors from these series are shown in Fig. **6**.

### Aminobenzimidazole Inhibitors

**Fig. (7):**
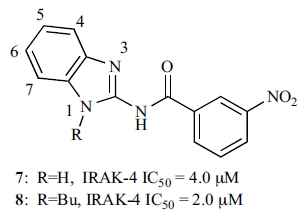
Initial IRAK-4 inhibitor hits from an aminobenzimidazole series.

A series of novel acyl-2-aminobenzimidazole inhibitors (**7** and **8** in Fig. **7**) were discovered as a result of high-throughput screening of a small molecule library against IRAK-4 kinase [59]. Comprehensive SAR studies carried out by Powers *et al*. resulted in significant improvement of potency as well as determination of the key structural features of the series.

The central amide functionality was found to be critical for IRAK-4 inhibition, since either removal of the amide carbonyl or replacement of the amide moiety with urea and sulfonamide groups resulted in a substantial reduction of IRAK-4 inhibition, as did the replacement of the free amide N-H with N-alkyl substitution. Modification of the benza-mide aryl ring with a limited set of substituents showed that some changes were allowed, but a 3-nitro group seemed to result in the highest potency. SAR around the benzimidazole group was explored on the 4-, 5-, 6-, and 7-positions, and the N was substituted with alkyl groups. An SAR study of the substituents on the ring demonstrated the importance of the benzimidazole ring. Improvements in potency were observed from the modifications on the 5-position and the N-alkyl substitutions.

**Fig. (8):**
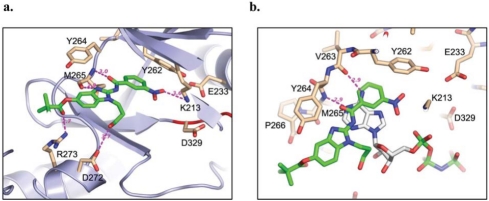
Inhibitor compound 1 binding in IRAK-4. **a)** the binding mode of 1 in the ATP-binding pocket of IRAK-4 (PDB code: 2NRU). **b)** 1 superimposed on the AMP-PNP complex structure of IRAK-4 (PDB code: 2OID).

Compound **1** (Fig. **6**) represents one of several potent inhibitors developed from the aminobenzimidazole SAR exploration by Powers *et al*. [39]. The cocrystal structure of IRAK-4 with **1** (PDB code: 2NRU) revealed the inhibitor bound at the ATP-binding site, with the 3-nitrobenzamide ring positioned towards the back and the benzimidazole ring at the front (Fig. **8a**). The binding of **1** to IRAK-4 is mediated through several hydrogen bonds and numerous van der Waals interactions. Compound **1** makes a hydrogen bond with the hinge region of IRAK-4 mainly through the amide carbonyl, which accepts a hydrogen bond from the backbone amide of Met265. The 3-position N of the benzimidazole ring also has a favourable hydrogen bond distance to the carbonyl moiety of Met265. The importance of the free amide N-H was investigated, and replacements of the amide N-H lost activity [59]. Together, the data suggested that the necessity of the free amide N-H may indicate a requirement for the inhibitors to be able to populate a tautomeric form via abstraction of this hydrogen [59]. In the back, the 3-nitrobenzamide ring forms a π-π stacking interaction with the gatekeeper Tyr262, with the nitro moiety forming a weak hydrogen bond with catalytic Lys213. In the middle, the N-propanol moiety points to the ATP ribose-binding area with its hydroxyl group pointing to Asp272 of helix αD. At the front, the trimethyl acetyl ester substitution at the 5-position of the benzimidazole ring points to the solvent-exposed region with its carbonyl moiety forming a hydrogen bond with Arg273 of helix αD. 

Comparison of the IRAK-4 complex structures with **1** (PDB: 2NRU) and AMP-PNP (PDB: 2OID) shows that **1** binds in partial overlap with the nucleotide, with its amide linker occupying the binding area of the adenine ring but extending more outward to the front of the ATP-binding area which ATP does not exploit (Fig. **8b**). The unique extended front area of IRAK-4 presents an appealing opportunity for the design of selective inhibitors. 

### Thiazole and Pyridine Amide Inhibitors

**Fig. (9):**
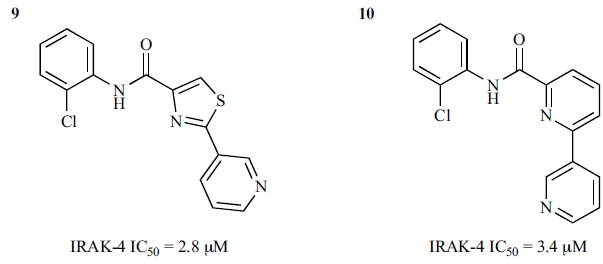
Initial thiazole amide IRAK-4 inhibitor and its pyridine amide analogue.

The thiazole amide **9 **(Fig. **9**) was identified as an initial hit from screening a small molecule library against IRAK-4 [60]. SAR studies around **9** carried out by Buckley *et al*. [60] resulted in a set of potent compounds with excellent solubility and good levels of exposure *in vivo*.

In the absence of cocrystal structural data, homology modelling and in silico docking were used to envisage the binding mode of **9** in IRAK-4. The thiazole amides were predicted to have the amide linker forming a key interaction to the hinge with the thiazole pyridine ring pointing towards the back and the substituted phenyl sitting at the front of the ATP-binding site. Initial SAR efforts were focused around the phenyl ring, for which a diverse set of commercially available phenyls were selected, including mono- and multi-substituted phenyls. The most active compounds from this effort were those having substitution at both the ortho- and the para-positions, such as **2** (Fig. **6**), which demonstrated significantly improved potency against IRAK-4 compared with the initial hit **9**. 

To facilitate the investigation of SAR around the other end of the molecule, i.e, the thiazole pyridine rings, the thiazole ring was replaced by another pyridyl ring. Compound **10 **(Fig. **9**) showed a similar potency to its thiazole analogue **9**. SAR around the terminal pyridine ring was found to be sensitive; the orientation and basicity of the nitrogen atom seemed important for modulating activity against IRAK-4. Pyrazoles showed improvement in potency against IRAK-4. The most active inhibitors, such as **3 **(Fig. **6**), were developed by combining the most promising substituted phenyls from the thiazole sub-series and the best terminal pyridine replacement from the pyridine sub-series. The authors also indicated that these potent inhibitors showed reasonable stability in rat and human microsomes, exposure in Lewis rats, and aqueous solubility [60]. 

### Imidazo[1,2-a]pyridine Inhibitors

Through a kinase project cross-screening effort of medicinal chemistry target compounds and advanced intermediates, a 2-aminopyrimidine-based imidazo[1,2-*a*]pyridine inhibitor **11** (Table **2**) from a JNK kinase project was identified as a potent inhibitor of IRAK-4 [62]. However, the aminopyrimidine-based scaffold is not novel as a kinase inhibitor; the aminopyrimidine moiety often forms “plug” (two hydrogen bonds) interactions with the hinge backbone of the ATP-binding site [65, 66]. To elucidate the novelty of this series as an IRAK-4 inhibitor, Buckley *et al*. performed in silico docking with homology modelling and surrogate crystal structure analysis together with preparation of some analogues [62]. This effort led to the identification of the imidazopyridine nitrogen atom N1, not the aminopyrimidine moiety as observed in other kinase projects, as the key structural feature of the series for the hinge interaction.

**Fig. (10):**
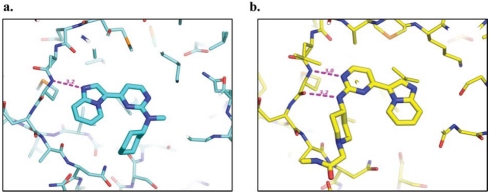
Cocrystal structures of aminopyrimidines in JNK-3. **a)** A more potent IRAK-4 compound: binding mode of **13** in JNK-3 (PDB code: 3CGF). **b)** A more potent JNK-3 compound: binding mode of **14** in JNK-3 (PDB code: 3CGO).

Figure **10a** shows the binding mode of the N-methyl capped **13** (IRAK-4: IC_50_ = 0.4 μM; JNK-3: IC_50_ = 3 μM) in a closely related homologous JNK-3 crystal structure (PDB code: 3CGF), which is consistent with the docking results from the IRAK-4 homology models [62]. While the nitrogen N1 atom of the imidazopyridine moiety serves as a hydrogen bond acceptor for the hinge backbone amide, the amino-pyrimidine sits in the ribose-binding area for ATP. A structurally related aminopyrimidine **14**, which is a more potent inhibitor for JNK-3 (IC_50_ = 270 nM) and less potent for IRAK-4 (IC_50_ = 3 μM), displayed an altered binding mode in a JNK-3 crystal structure (Fig. **10b**) (PDB code: 3CGO). Here the inhibitor adopted the typical binding mode often observed in other kinases, with the aminopyrimidine being the key pharmacophore for the hinge interactions.

**Fig. (11):**
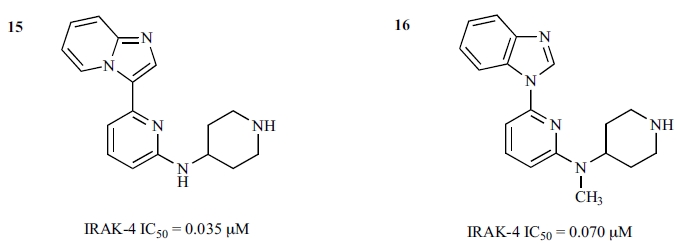
Lead compounds of the imidazo[1,2-*a*]pyridino-pyridine and benzimidazole pyridine series.

Following the establishment of the key structural features for the series, Buckley *et al*. carried out more investigations of SAR around the lead compound **11** [61]. This effort brought forth two more lead compounds, **15** (an imidazo[1,2-*a*]pyridino-pyridine) and **16** (a benzimidazole-pyridine), as the more preferred scaffolds for optimization (Fig. **11**). The new lead series was developed to remove the initial amino-pyrimidine motif and its associated potential promiscuity. The SAR around the new leads generated highly potent IRAK-4 inhibitors, such as **4**, **5**, and **6** (Fig. **6**). While these compounds were reported to have good cellular TNF-α inhibition, good drug-like properties, and encouraging *in vitro* DMPK profiles, some degree of off-target activity was still evident. As these molecules still have low molecular weights (300-350 Da), the series remains an interesting class for further optimization and development into potent and selective inhibitors. 

### Imidazo[1,2-*b*]pyridazine and Indazole Inhibitors

**Fig. (12):**
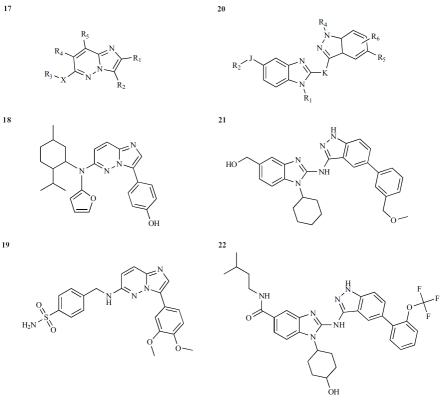
Imidazo[1,2-*b*]pyridazine and indazole IRAK-4 inhibitors.

Imidazo[1,2-*b*]pyridazines and indazoles were disclosed in two recent patents published by Biogen Idec as IRAK-4 modulators for treatment of some inflammatory, cell proli-ferative, and immune-related disorders [58, 64]. Apparently, various compounds were developed around the two key formulas, **17** and **20** (Fig. **12**). These compounds were reported to exhibit IC_50_ values of less than 20 μM, and some had IC_50_ values of less than 10 nM. However, specific IC_50_ values for these compounds are not shown in the patents. 

In summary, several novel scaffolds suitable for the development of IRAK-4 inhibitors have been identified. They potently inhibit IRAK-4 kinase activity and have reasonable cellular potency and physicochemical properties. Structural analysis suggests that there are plentiful opportunities for further improvement of potency, selectivity, and other physicochemical properties. Further optimization of these promising compounds may lead to new therapeutic agents for inflammatory diseases. 

## CONCLUSIONS

IRAK-4 is a critical molecule mediating signals in both innate and adaptive immune responses. Though characterized functionally as a serine/threonine kinase, the crystal structures revealed that IRAK-4 contains characteristic structural features of both serine/threonine and tyrosine kinases, as well as additional novel attributes, including the unique tyrosine gatekeeper residue. The ATP-binding site in IRAK-4 has no deep pocket in the back but has a featured front pocket. This uniquely shaped binding pocket provides an excellent opportunity for designing IRAK-4 inhibitors which confer high selectivity. 

The development of IRAK-4 kinase inhibitors has generated several novel classes of potent binders in the ATP-binding pocket. Apparently, all of them are still in the early preclinical stage. The potential for clinical utility of IRAK-4 inhibitors in inflammation continues to change as the role of IRAK-4 in the complex signaling cascade is further elucidated. As an example, early studies suggest that the IRAK-1 kinase activity might be a negative feedback signal [67], and it therefore seems desirable to avoid any IRAK-1 activity in IRAK-4 compounds. However, another study communicated by Roche [63] suggested that pharmacologic inhibition of both IRAK-4 and IRAK-1 might be necessary to block pro-inflammatory cytokine production. The compounds they described and their derivatives will hopefully lead to a conclusive elucidation of the relative contribution and the interdependencies of IRAK molecules in TIR signaling and inflammatory processes.

## Figures and Tables

**Table 1.** Structures of Human IRAK-4 Kinase Domain Deposited at the RCSB Protein Data Bank

PDB code	Ligand	Resolution (Å)	Phosphorylation Site	Refs
2O8Y	None (Apo)	2.4	pT342, pT345	
2OIB	None (Apo)	2.0	pT342, pT345, pS346	[40]
2OID	AMP-PNP	2.3	pT342, pT345, pS346	[40]
2OIC	Staurosporine	2.4	pT342, pT345, pS346	[40]
2NRY	Staurosporine	2.2	pT345	[39]
2NRU	Benzimidazole	2.0	pT345, pS346	[39]

**Table 2.** Inhibition of IRAK-4 by Imidazo[1,2-*a*]Pyridine Analogs Identified from a Cross-Screening of other Kinase Projects